# Exploring adolescent academic stress in the digital and urban age: a mixed-methods study from CIT to checklist validation

**DOI:** 10.3389/fpsyg.2025.1692113

**Published:** 2025-11-12

**Authors:** Ruobing Wang, Shengqin Yang, Xiao Xu, Shuaishuai Mi, Na Hao

**Affiliations:** 1 Faculty of Education, University of Macau, Taipa, Macao SAR, China; 2 Xianda College of Economics & Humanities, Shanghai International Studies University, Shanghai, China; 3 School of Physics and Electronics, Shandong Normal University, Jinan, China; 4 School of Art & Design, Guangzhou College of Commerce, Guangzhou, China; 5 Faculty of Humanities and Arts, Macau University of Science and Technology, Taipa, Macao SAR, China

**Keywords:** academic stress, adolescents, mobile Internet, urban community, weak ties, ecological systems theory, critical incident technique, checklist validation

## Abstract

**Introduction:**

In recent decades, middle and high school students have been experiencing increasing levels of academic stress. The reason may be rapid urbanization and the widespread use of the Internet, which have expanded students’ environments from the confines of family and school to the open community and cyber world. Greater exposure to academic information and social interactions may contribute to heightened stress levels. However, the underlying mechanisms remain underexplored. Furthermore, the current taxonomy of academic stress conflates self-stress with stress arising from social events, resulting in a misalignment between theoretical frameworks and measurement scales.

**Materials and methods:**

The mixed-methods study explored stressful academic events among adolescents using the critical incident technique and validated a corresponding scale (Adolescent Stressful Academic Events Checklist, ASAEC). The study was conducted during the spring 2025 semester. In the qualitative phase, 84 participants, including teachers, parents, and students, were interviewed. Data were coded, member-checked, and analyzed to identify common stressful academic events. Based on these findings, a checklist of adolescent academic stress was developed, and its reliability and validity were examined through a survey of 453 adolescents.

**Results:**

(1) Qualitative: The study categorized critical incidents from three dimensions (event topics, social actors, and interaction ways). A total of 540 critical incidents revealed common sources of academic stress related to enrollment, rivalry, career, working hard, intelligence, and social. These events were associated with various social actors, including neighbors, kinsmen, friends of parents, non-classmate peers, netizens, news media, self-media, parents, teachers, and classmates. Participants reported experiencing stress not only through direct interactions but also through mediated, observing, and distal interactions. (2) Quantitative: Both EFA and CFA supported a single-factor structure for the checklist. The Item Response Theory (IRT) model demonstrated strong psychometric properties, including high reliability, good model fit (as indicated by RMSEA, CFI, and TLI), and appropriate item parameters, infit indicators, and average information measures. ASAEC showed significant correlation with the hypothesized variables, confirming its criterion-related validity.

**Conclusion:**

(1) Newly identified stressful academic events were found to be correlated with urban and online communities, and all related items were significant in the survey. These findings highlight the importance of considering the impact of digitalization and urbanization on academic stress. (2) Weak ties and new interaction ways were found to contribute to stressful academic events, while the checklist exhibited a single-factor structure. The findings suggest that the proposed three-dimensional framework requires further empirical validation. (3) ASAEC provides a reliable tool for assessing adolescent academic stress from an ecological perspective, facilitating more effective management that accounts for various interactions with a wide range of social actors.

## 1 Introduction

### 1.1 Background

Academic stress (AS) is a growing concern among secondary school students worldwide, with significant implications for their mental health and overall well-being. Excessive AS has been linked to anxiety and depression, making it a critical issue within education systems globally (Zauderer, 2023). While moderate levels of AS can motivate learning, the increasing intensity of academic demands has led education systems to implement measures aimed at alleviating overstress (CPC and SC, 2021). Despite these efforts, the problem persists, with recent studies reporting severe outcomes such as increased TikTok addiction and rising student suicide rates (Chao et al., 2023; Song X. Z. et al., 2023). These findings suggest that current interventions may overlook key factors contributing to AS in today’s rapidly changing social environment.

A key perspective in understanding and addressing AS involves identifying the specific stressful academic events (SAEs) that contribute to its development. SAEs can be defined as stimulating situations that directly trigger AS in students. Unlike behavioral, physiological, or psychological responses to AS—which vary based on individual personalities, social resources, and coping strategies—SAEs offer a more reliable target for assessment and intervention (Kaynak et al., 2023). Identifying common SAEs within specific social contexts can provide valuable insights for both educators and policymakers. However, SAEs are not static; they evolve in response to broader societal changes (Cai et al., 2022), such as societal and technological advancements.

In recent decades, urbanization and the widespread adoption of mobile Internet have dramatically transformed adolescents’ social environments (Marquez et al., 2024; Moreno et al., 2021). Urban communities, which have replaced traditional village consanguinity networks, have become the dominant social structure in countries such as China (Song Y. K. et al., 2023). At the same time, social media platforms and online news have become integral parts of adolescents’ lives, criticized for facilitating cyberbullying, amplifying societal pressures, and spreading anxiety (Hamm et al., 2015; Shensa et al., 2018; Moreno et al., 2021). Together, these changes have created a new environment where adolescents are increasingly influenced by urban communities and online interactions.

The AS scales have largely focused on social actors in family and school that contribute to AS (Garcia-Ros et al., 2018; Hosseinkhani et al., 2020; Kaynak et al., 2023). However, these scales often overlook the influence of acquaintances such as neighbors, kinsmen, parents’ friends, netizens, and the media. These overlooked factors highlight the need for a more comprehensive understanding of AS in the urban communities.

Adolescents frequently attribute their AS to personal inadequacies or internalized expectations, overlooking how public opinion, social media, and indirect interactions shape their ideas (Shensa et al., 2018; Nagabharana et al., 2021). Previous scales defined “self” stress as parallel to that of parents, teachers, and peers as social actors (Ang and Huan, 2006; Xu et al., 2010). This taxonomy, simply explained, describes AS without direct external sources as “self” stress, failing to locate an individual within their social exosystem. Furthermore, from the perspective of taxonomy, AS scales failed to separate the dimensions of stressful events, social actors, and interaction ways, which resulted in their indicators being named chaotically. Besides the issues mentioned above, some scales categorized “self” stress and “peer” stress parallel to stressful events (Sun et al., 2011; Hosseinkhani et al., 2020), which made the construct lacking well-identified criteria, necessitating a theoretical re-examination.

### 1.2 Research aims

The research aims to explore typical SAEs in China, providing a broader understanding of AS among middle and high school students within a digital and urban context. While previous research has primarily focused on SAEs within family and school environments, this research expands the scope by examining SAEs that arise in any social context, particularly those related to urban or Internet communities.

The research seeks to challenge the conventional understanding of “self” as an internally driven indicator from the dimension of social actors. Instead, it argues that what students perceive as “self” stress is often shaped by external influences, such as societal norms, cultural expectations, and pervasive messaging from media and public discourse. These external influences are internalized over time, leading students to believe that high expectations or academic success are their natural duties, while neglecting the societal origins of these beliefs. Compared to SAEs that students interact with specific objects, “self” stress arises from long-term interactions that cannot be attributed to a particular social actor. From this perspective, the “self” stress is named “distal interaction” to highlight the long-term, hidden impact of social structures, media, and public opinion on students’ academic stress. The distal interaction is categorized into the dimension of interaction ways, parallel to direct interaction, mediated interaction, observing interaction.

To address the issue of conflating event topics, social actors, and interaction ways in previous scales, this study distinguishes these three dimensions. The qualitative data were coded accordingly. In light of the limited consideration of urbanization and Internet influence in previous scales, this study aims to develop a new scale (Adolescent Stressful Academic Events Checklist, ASAEC) comprising the SAEs identified in the qualitative phase. The quantitative data can also provide evidence on whether SAEs related to urban communities and online interactions are important by examining whether the new items are significant or not in the checklist. The checklist can also be used to explain the issue of ill-identified dimensions.

In order to realize the research aims, the research has six questions: (1) What are the common stressful academic events (SAEs) experienced by adolescents in the digital and urban contexts? (2) Are the SAEs related to the urban community and online interactions significant in a checklist that consists of all SAEs? (3) Which social actors are attributed to these SAEs besides those actors in the family and school? (4) How do the social actors interact with adolescents to bring these SAEs? (5) Do the SAEs have a dominant dimension to explain their construct structure? (6) Is the Adolescent Stressful Academic Events Checklist composed of identified SAEs a reliable and valid measure of adolescent academic stress?

### 1.3 Literature review

#### 1.3.1 SAE topics

The literature provides robust evidence supporting the existence of various SAE topics. Some psychological scales have identified important SAE factors. Due to reliance on EFA methods, the labels attached to the factors in psychological scales may not precisely reflect the nature of the events. However, some qualitative research has provided vivid details of students’ SAE experiences and feelings. The similar findings of qualitative and quantitative literature provide complementary evidence for the existence of the following SAE topics.

##### 1.3.1.1 Expectation from others

Similar insights have emerged from numerous scales developed across different countries, including Iran (teachers’ expectations; Hosseinkhani et al., 2020), Turkey (high parental expectations; Kaynak et al., 2023); East Asia (academic expectations; Ang and Huan, 2006), China (others’ expectations; Chen, 2004; Lin, 2014; enrollment in high schools and universities; Liu et al., 1997). Qualitative research further supports these findings in India (achieving excellent academic performance; Nagabharana et al., 2021; parental specific expectations for achievement; Rao, 2008), Iran (perfectionism of the families; Hosseinkhani et al., 2019), and the USA (course expectations; Boyle, 2020; perfectionism; and community culture emphasizing “college as the end goal and status symbol,” “grades as a priority,” and “climbing the ladder of success”; Cusato, 2022).

##### 1.3.1.2 Expectation of their own academic performance

Similar insights emerged from applicable scales in the USA (academic self-worth/self-esteem/self-concept; Helms and Gable, 1990), East Asia (academic expectations for oneself; Ang and Huan, 2006), and China (self-expectation; Sun et al., 2011; Lin, 2014; academic goals; Chen, 2004; Wang, 2008). However, there is no qualitative evidence for the SAE.

##### 1.3.1.3 Troubles about their academic performance

It has been documented in scales from the USA (academic performance/achievement; Helms and Gable, 1990), China (worrying about grades, or despondency toward academic behaviors; Sun et al., 2011; frustration, exam failure, or failing to be chosen as the best performers; Liu et al., 1997; frustration; Wang, 2008; academic prospects; Lin, 2014; negative cognition; Lin, 2014). Qualitative research also reported the same finding in India, where participants had no active attitudes or beliefs about academic standards (Rao, 2008).

##### 1.3.1.4 Worrying about their future education and employment

The SAE represents a significant source across scales, from China (future education and employment; Sun et al., 2011) to Spain (a future-oriented perspective; Garcia-Ros et al., 2018) and Iran (future uncertainty; Hosseinkhani et al., 2020). However, there is no qualitative evidence for the SAE.

##### 1.3.1.5 The competition with others

Scales have shown that it also functions as a significant stressor in China (competition among classmates; Sun et al., 2011; competition; Chen, 2004; Wang, 2008; academic competition; Lin, 2014), as well as in Iran (academic competition; Hosseinkhani et al., 2020). However, there is no qualitative evidence for the SAE.

##### 1.3.1.6 Others’ comparison behavior

Similar insights were gained from applicable scales in China (Xu et al., 2010) and from qualitative research in India, where parents compared their children to others (Rao, 2008), as well as in Iran, where parents compared adolescents with others (Hosseinkhani et al., 2019).

##### 1.3.1.7 Others’ bad attitudes in school

It is another well-documented topic in scales from the USA (teachers’ attitudes toward students and classmates’ feelings toward them; Helms and Gable, 1990), Spain (what my classmates think about them, conflicting and working with classmates; Garcia-Ros, et al., 2018), and Iran (teacher neglect or rapport, difficulties fitting in, and disagreements with classmates; Hosseinkhani et al., 2020). Qualitative research further highlights related stressors in India, such as malicious behavior from teachers (Nagabharana et al., 2021), and in the USA, including conflict/bullies/violence, or feeling judged/misunderstood (King, 2017).

##### 1.3.1.8 Social problems with peers

Qualitative research indicates that it is a significant category in Iran, characterized by a lack of integrity among friends and peers, as well as being mocked by friends (Hosseinkhani et al., 2019). Similarly, in the USA, SAE is associated with social interactions with friends and peer relationships (Boyle, 2020).

##### 1.3.1.9 Parents’ interference in academic life

Similar insights have emerged from applicable scales in Turkey (psychological pressure from parents, restrictions by their parents to increase their success; Kaynak et al., 2023), Iran (home life and parents’ involvement in academic life; Hosseinkhani et al., 2020), and China (parents care about my grades; Sun et al., 2011). Qualitative research describes similar patterns, including in India (parents put AS on their children or controlled the study environment; Rao, 2008) and in the USA (loss of freedom and autonomy) (King, 2017).

##### 1.3.1.10 Academic overload

It is frequently identified in scales from China (studying workload; Sun et al., 2011; task burden; Liu et al., 1997; academic tasks; Chen, 2004), Spain (academic overload; Garcia-Ros et al., 2018), and Iran (learning tasks; Hosseinkhani et al., 2020). Qualitative research also reported this finding in India (busy schedules; Rao, 2008), Iran (the mass of homework and exams; Hosseinkhani et al., 2019), and the USA (time spent preparing for courses; Boyle, 2020; schedule overload due to difficulties balancing personal and school life; Cusato, 2022; facing an overwhelming amount of work or homework; King, 2017).

##### 1.3.1.11 School infrastructure and environment

Poor school infrastructure and inadequate facilities have been linked to academic stress in China, including a negative environment (Chen, 2004), an unfavorable external environment (Wang, 2008), and a subpar learning environment (Lin, 2014). Qualitative research also reported findings in India, where insufficient school infrastructure and a lack of basic facilities, such as canteens, were noted (Nagabharana et al., 2021). Similar issues have been reported in Iran, particularly in the school environment (Hosseinkhani et al., 2019).

##### 1.3.1.12 School regulations

Stress caused by school policies and regulations has been identified in scales from Iran (school regulations; Hosseinkhani et al., 2020) and China (behavioral demands; Chen, 2004). Qualitative research has also reported findings in India (time stress caused by improper school scheduling; Nagabharana et al., 2021) and the USA (labeling and tracking students based on the number of rigorous courses they take and having to choose certain courses without interest due to such labels; Cusato, 2022).

##### 1.3.1.13 Students’ disagreement with academic rules

Students’ dissatisfaction with academic systems and rules has been examined in scales from Iran (educational system; Hosseinkhani et al., 2020). Qualitative research in India has also reported similar findings, highlighting frustration with a perceived disconnect between coursework and real life, rendering learning experiences meaningless, as well as dissatisfaction with rigid and unreasonable rules that use exams to determine students’ entire futures (Rao, 2008).

##### 1.3.1.14 Financial problems

Similar insights emerged from an applicable scale in Iran (financial pressure; Hosseinkhani et al., 2020) and corresponding qualitative research (inappropriate family situations; Hosseinkhani et al., 2019). However, other research teams did not find a similar topic.

##### 1.3.1.15 Shadow education

Similar insights were obtained from qualitative research in Iran (educational assistance classes; Hosseinkhani et al., 2019) and the USA (participation in extracurricular activities; Boyle, 2020). However, there is no quantitative evidence for the SAE.

##### 1.3.1.16 Social media

Similar insights were gained from qualitative research in the USA, where participants reported wasting time on social media with friends and family, worrying that others disliked their posts (Boyle, 2020), comparing themselves to edited versions of peers’ lives on social media, and noticing that they were excluded from social events (Cusato, 2022). However, there is no quantitative evidence for the SAE.

#### 1.3.2 Social actors in SAEs

The AS arises from the interaction between individuals and their environment. Students process and evaluate the SAEs in cognition when perceiving threats or challenges in the environment, and AS occurs when they feel incapable of handling them (Lazarus, 1997). Consequently, AS is originally caused by stimuli embedded in social interactions, and such stimuli may originate from various sources of information. It is necessary to learn who adolescents may contact for information.

##### 1.3.2.1 Parents and teachers

For adolescents, significant others such as parents (Kemper, 1963) and teachers (Engle and Szyperski, 1965) play a vital role in shaping individual development and well-being. Parents and teachers are particularly influential in the socialization and self-concept formation process of adolescents. Their attitudes toward academic achievements significantly affect how adolescents perceive their academic responsibilities, thereby acting as a source of AS (Ang and Huan, 2006).

##### 1.3.2.2 Peers

Peers constitute the major reference group for adolescents. These reference groups can be formal (i.e., class and school), informal organizations, or conceptual roles (i.e., children, students, and adolescents). Students tend to imitate the ways their reference groups engage in social interactions and strive to perform ideal roles aligned with group norms. They evaluate their own performance through peer feedback and by comparing themselves to these norms (Kelley, 1952). Such peer pressure encompasses AS. In scales and qualitative research, there is evidence that SAEs can be attributed to classmates (Xu et al., 2010) and peers (Boyle, 2020).

##### 1.3.2.3 Weak ties

Granovetter’s (1973) theory suggests that weak ties, characterized by infrequent communication and loose connections, far outnumber strong ties and exert subtle yet widespread influences on individuals within social networks. Parents, teachers, and classmates, due to their close social proximity and frequent interactions with students, are represented as strong ties. These strong ties significantly influence students’ AS, but weak ties might also play a role. Weak ties for adolescents include neighbors, kinsmen, non-classmate peers, and netizens. Although interactions with these weak ties are infrequent, their sheer volume makes them a significant factor in students’ social cognition. In qualitative research, there is evidence that SAEs can be attributed to the community (Cusato, 2022) and the school’s regulatory culture (Hosseinkhani et al., 2020; Nagabharana et al., 2021). There is seldom quantitative research that uses weak ties as a factor in AS research, but there has been strong evidence that weak ties are important for information spreading and identity recognition (Lieberman and Schroeder, 2020; Sprecher, 2022).

Mainstream media continuously reinforce the notion that academic degrees are a prerequisite for employment (Spence, 1973) and that higher academic achievement corresponds to higher social status (Young, 1958). In traditional Chinese cultural values, the concepts “All pursuits are inferior to studying” and “Mastering STEM makes you fearless in the world” are deeply ingrained. By cultivating public opinions that legitimize the education system and maintaining social consensus around education (Gerbner and Gross, 1976), the media’s promotion of concepts such as “top scorers,” “academic champions,” and “admission rates” may serve as sources of AS for middle and high school students. In qualitative research, there is evidence that SAEs can be attributed to Internet social media (Boyle, 2020; Cusato, 2022).

##### 1.3.2.4 Self

Through prolonged exposure to external information and feedback from social interactions, students gradually internalize (Deci and Ryan, 1985) norms about being a “good student” and the belief that studying is of extreme importance. They set personal goals and feel stressed when they fail to meet these goals. According to the Looking-Glass Self Theory (Cooley, 1902), students imagine how others perceive their academic performance in social contexts, which may lead to self-doubt, shame, and associated emotions. Such AS is endogenous and does not require external stimuli to manifest. However, the mechanism that generates endogenous AS is rooted in prolonged and repeated exposure to external stimuli in their early lives. The mainstream ideas mold adolescents’ cognition stably, slowly, and covertly, putting them under chronic AS. It may have a greater influence than a drastic conflict with parents or teachers (James et al., 2023), acting as the root cause of AS. An existing scale lists “self” as a factor parallel to parents, teachers, and classmates (Xu et al., 2010).

In certain contexts, subcultures reject mainstream educational values and develop the belief that schooling is futile. Among such groups, students may adopt a goal of “hustling in society,” viewing skipping classes or defying teachers as admirable. And they believe their career paths are predetermined, rendering academic effort irrelevant (Willis, 1978). These students exhibit lower levels of AS. Conversely, some students resist “delinquent” subcultures and aspire to self-improvement, fearing the labels of “nerds” or “bookworms” and being ostracized by their peers (Harding, 2012). These students face a conflict between the need for social belonging and long-term personal development. Such role conflict heightens AS. In qualitative research, there is evidence that the feeling of disagreeing with school rules is associated with AS (Hosseinkhani et al., 2020; Rao, 2008).

#### 1.3.3 Interaction ways in SAEs

The dimension of interaction ways is a reorganization of social actors. The research conceptualized the dimension from the perspective of ecological systems theory in order to explain the contradiction between the theory of external stimuli and the existence of self-stress.

**Figure 1:**
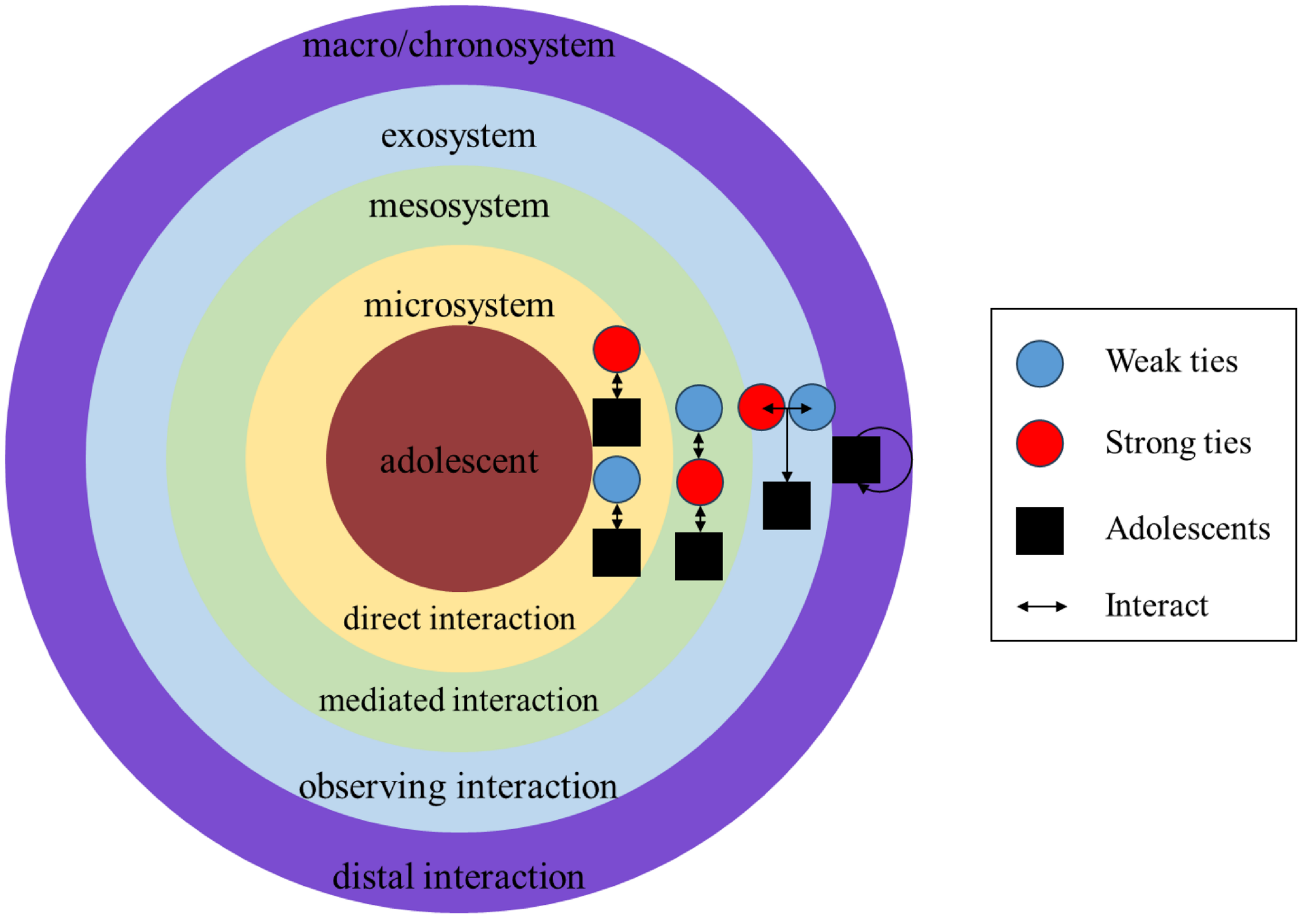
SAEs framework in layers.

The ecological systems theory posits that individuals are placed in environments comprising multiple layers of systems. The closest system to individuals is the microsystem, in which individuals interact directly with others. The mesosystem influences individuals through the interaction among elements in the microsystem. An exosystem refers to the environment in which individuals seldom interact directly but are still influenced indirectly. The macrosystem refers to the framework that typically operates within the overall environment in which individuals live, encompassing culture, which includes social structure, laws, and policies. Finally, the chronosystem emphasizes that the experience has a strong connection to individuals’ development today (Bronfenbrenner, 1977; Cusato, 2022). The research integrates the above-mentioned SAEs and explains the interaction of AS based on this theory (see Figure 1).

##### 1.3.3.1 Microsystem

Parents, teachers, and classmates interact directly with adolescents in family and school settings, and they are the major social actors in the microsystem. However, prior research focused on students’ interactions with strong ties, ignoring peers, neighbors, kinsmen, or netizens. Moreover, the research explored whether students’ interactions with weak ties trigger AS.

##### 1.3.3.2 Mesosystem

According to the literature, the interaction between strong ties and weak ties can trigger students’ AS, and the SAE would be attributed to the actor who directly interacts with students. For example, the SAE of “parents’ opposing children’s playing with classmates with low academic achievements, triggering their social conflicts with their classmates” is attributed to classmates (Rao, 2008). However, conflicts among students may reflect the interaction in the mesosystem, which means that the role of parents cannot be neglected. From the perspective of ecological systems theory, both parents and classmates would be considered related social actors in the mesosystem. Such interaction ways, which involve more than one social actor, are distinguished from direct interaction in the microsystem and serve as a category in data processing. It is called “mediated interaction,” which refers to the phenomenon that a third party triggers adolescents’ AS by influencing someone’s interaction with adolescents. The mediated interaction occurs within the mesosystem.

##### 1.3.3.3 Exosystem

Students do not need to experience an event to feel stressed. The theory of observational learning has proven that individuals can observe others’ behaviors to acquire relevant behaviors or attitudes (Bandura, 1965). Research about PTSD also found the damage to mental health by merely being a witness to catastrophes (Bliese et al., 2008). Similarly, students’ observing SAEs should be considered in exosystems. For instance, when peers discuss exams and enrollment (Cusato, 2022), students may be influenced, even if the SAE is not related to them. The interaction among others influences students’ AS through students’ observation; therefore, the research refers to it as “observing interaction” and uses this term in the data processing. The observing interaction happens in the exosystem.

##### 1.3.3.4 Macrosystem and chronosystem

The culture, laws, and educational institutions shape the social mainstream’s views on students’ academic achievements (Foucault, 1975). The majority of citizens recognize the mainstream ideas to some extent, acting as a voice in the chorus of mainstream ideas. Students stay in the chorus for a long time and finally internalize the norms, believing that the AS comes from their own expectations and needs. When students feel stressed without any external stimuli, the so-called self-stress is a mirror of the long-term discipline of mainstream ideas, i.e., the combination of macrosystem and chronosystem. It is challenging to determine the details and sources of mainstream ideas underlying self-stress; however, the existence of self-stress reflects the influence of both the macrosystem and the chronosystem. Thus, self-stress is referred to as distal interaction and is used as a category in data processing.

## 2 Materials and methods

The study was approved by the Shandong Normal University’s Ethics Committee (Approval No. 2025-03-05). The research team contacted participants through referrals from their teachers and provided oral descriptions of the research objectives and content. Interviewees were thoroughly briefed on the research purpose, the voluntary nature of their participation, their right to withdraw at any time without penalty, and measures taken to ensure anonymity and confidentiality. The participants of the questionnaire survey also reviewed the informed consent form in the Wenjuanxing system and indicated their agreement to participate by checking a “consent to participate” box. All data were anonymized and stored securely in password-protected databases, with no personally identifiable information linked to the responses.

### 2.1 Critical incident technique

#### 2.1.1 Data collection

**Figure 2:**
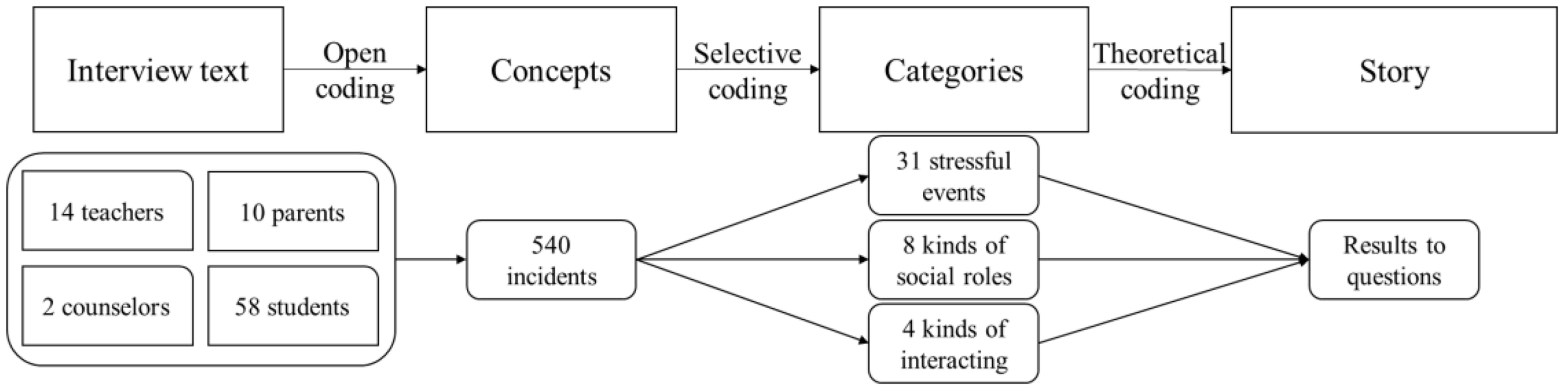
Research process.

The Critical Incident Technique (CIT) refers to an approach where researchers collect entire and observable human activities in contexts where goals and intentions are clear. Researchers identify critical incidents that can infer and predict people’s behaviors, categorize them, and extract potential value to address practical issues. Originating from Flanagan (1954), CIT focuses on the specific procedures and principles used, as well as which critical groups to study, to effectively collect data. To gain an understanding of SAEs among middle and high school students, collecting critical incident data has been examined as an effective method (Nguyen et al., 2013; Hosseinkhani et al., 2019). The research employed a structured interview similar to the CIT used to collect data (see Figure 2).

##### 2.1.1.1 Interviewer

Following the norm of CIT, two collaborators will act as interviewers to minimize bias in the recall and recording of incidents. The two interviewers have over 10 years of teaching experience, making them well-versed in the education system and qualified to conduct interviews. They are teachers at two schools, so the researcher has access to contact the participants. Their roles in the school can also help participants trust them during interviews. The interviewers are trained to ensure the quality of their interviews. The training includes an online introduction to the literature on AS, the research’s purpose, the reasons for interviewing different kinds of participants, the interview procedure, time management, and precautions (listening without comments, probing when necessary, and avoiding suggestive words).

##### 2.1.1.2 Interviewee

Following the norm of CIT, qualified participants should be involved in the sample. The critical incident technique (CIT) should collect more than three critical incidents from each participant if the empirical quality is to be considered acceptable (Watkins et al., 2022). Additionally, 100 incidents are considered adequate for grounded theory (GT) to be coded with good accuracy (Lockwood, 1994). Therefore, the sample should consist of more than 33 participants.

The research recruited 84 participants through theoretical sampling, with participants from a middle school (grades 7–9) in Fujian Province and a high school (grades 10–12) in Jiangsu Province (see Table 1). The interviewees should be individuals who play important roles in adolescents’ academic lives and can provide information from diverse perspectives. For that purpose, the interviewees included students, teachers, and parents. Specifically, the teachers included headteachers, subject teachers, and psychological counselors. They can reveal students’ AS from different perspectives. The headteachers and subject teachers can observe students in teaching and learning contexts as educational practitioners. The counselors can observe students in clinical and private contexts as psychological experts. Parents can observe students in family contexts as intimate but authoritative others. Meanwhile, students could reflect on their metacognition about their daily experiences.

**Table 1:** Participants.

Variables		Head teacher	Subject teacher	Psychological counselor	Parent	Student
Grade	7	1	1	–	2	4
	8	1	1	–	2	4
	9	1	2	–	2	5
	10	1	1	–	1	2
	11	1	1	–	1	2
	12	2	1	–	2	39
Male | female		3/4	3/4	1/1	5/5	31/27

To ensure that the data provided by the interviewees were typical and rich, the research selected participants based on specific criteria. The teachers interviewed should have at least 3 years of teaching experience. Parents should have mutual trust with the school and be vigilant about their children. The students should be at an average level in both academic achievement and social activities, of both gender. To accurately reflect the academic life of different stages, the interviewees covered grades 7 through 9 in middle school and grades 10 through 12 in high school. Specifically, because the college entrance examination (CEE) is important in China, making grade 3 students in high school face the most severe AS, the research oversampled a grade 3 class in high school.

##### 2.1.1.3 Interview process

One interviewer met with one participant in the teacher’s office each time, starting with informed consent, which included notification of the research topic and the use of sound recording. Then, the interviewer asked several questions in sequence, and the participants responded with relevant examples from their experience. Each interview lasted approximately 20 min, and the interviewer finally acknowledged the interviewee’s participation.

To identify typical SAEs, it is suitable for each participant to report on critical incidents, which would provide the most useful information. The questions designed using CIT are most conducive to collecting data that meet this research aim (Burnett and Fanshawe, 1997). The research employed deliberate and repeatedly revised interview outlines (see Appendix 1), allowing each participant to provide as much useful information as possible. Before the main questions, the outlines provide some warm-up questions, making the interviewees recall adolescents’ daily lives, overall AS status, social relationships, and electronic device use to arouse their related experiences and relax; then the outline turns to the research aims to learn how adolescents’ AS is influenced by outside factors. To answer the research questions, the outline employs a series of questions, including those from parents, teachers, classmates, peers, neighbors, those who live in the vicinity, kinsmen, strangers on the Internet, news sources, and self-media. The outline allows the interviewers to probe after any question to clarify the meaning of the interviewee’s prior words.

#### 2.1.2 Data analysis

The research aim is to identify typical SAEs by focusing on the commonalities reported by participants, rather than seeking differences among cases or exploring the impact of one experience on another. It is then necessary to abstract participants’ statements into codes and categories of SAEs (Parikh et al., 2019). To ensure the rationality of the generated categories, it is essential to clarify the definitions, similarities, differences, and relationships among the categories and to align the coding and categories with the original statements through multiple adjustments. The Classic Grounded Theory (CGT), which constructs theory through continuous comparison, aligns with this requirement.

While CIT emphasizes the details of data collection, it is somewhat ambiguous regarding how to analyze the data. To better utilize the data gathered, CGT can serve as a suitable complement to CIT, which has been conducted in other research (Watkins et al., 2022). CIT and CGT are constructivist paradigms that generate findings from evidence inductively rather than testing hypotheses deductively. CGT places greater importance on obtaining credible theoretical outcomes through specific coding and analysis procedures. In such a combination, CIT provides a structured framework for collecting data, while CGT offers a more rigorous and systematic procedure for analyzing data and generating theory. This integration creates a coherent whole for data collection and analysis, leveraging the strengths of both CIT and CGT. The combination can be used in a specific research context, and the SAE is such a context.

The research followed this process to analyze the data (see Figure 2). The interviewers recorded audio during the interviews. The interview recordings were transcribed into text, forming a text database for each participant. The first step is open coding, which means generating concepts that can be managed from text. In the research, the “concepts” should be critical incidents. The second step is selective coding, in which the concepts are categorized. This means that similar critical incidents were classified into the same category of SAEs, and these critical incidents were also determined to be related to specific social actors and interactions. The third step involves theoretical coding, where the researcher integrates categories into a readable “story” that combines SAEs, social actors, and interactions. The story is followed by one or two typical interviewer statements that help enrich the evidence details.

In the open coding, the interview text was refined to 540 critical incidents identified, with an average of 7.5 critical incidents per participant, which was higher than the general level (3 incidents per participant) of CIT technical requirements (Lockwood, 1994; Watkins et al., 2022). This corroborated that each participant provided adequate information, and the data were unbiased, reflecting typical SAEs.

**Figure 3:**
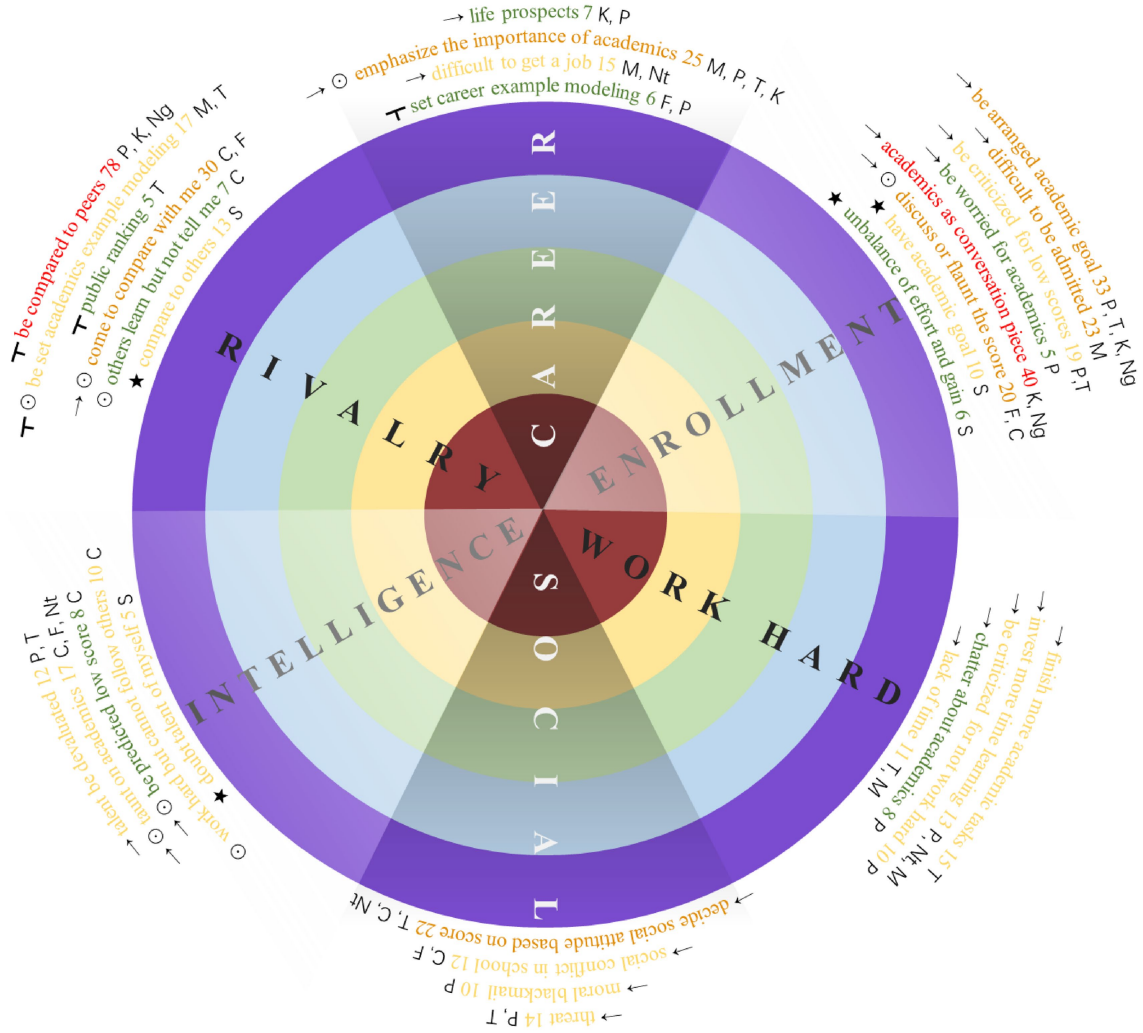
Brief results of the qualitative phase.

In selective coding, 31 SAEs are recognized through induction, and they are further categorized into six abstract categories. The number of critical incidents related to each SAE is presented in Figure 3. The 540 incidents were identified as relating to various social actors (i.e., parents, teachers, kinsmen, media, neighbors, people living in the vicinity, classmates, non-classmate peers, and netizens) and interaction ways (i.e., direct, mediated, observing, or distal interaction). The number of critical incidents related to each social actor and interaction is presented in Table 2. Most critical incidents can be recognized, while a few cannot due to their vague meanings, so the sum of the SAEs or interactions is less than 540. Considering that one critical incident can relate to more than one type of social actor (mediated interaction), the sum of social actors exceeds 540. In theoretical coding, if one SAE contains more than three critical incidents related to a single category of social actor and interaction, it would be labeled to that category.

**Table 2:** Results of theoretical coding.

Social relationships				Interaction	
Categories	Times	Categories	Times	Categories	Times
Parents	117	Media	54	Direct	326
Non-classmate peers	108	Kinsmen	53	Mediated	89
Classmates	84	Neighbor	45	Observing	67
Teachers	68	Netizens	28	Distal	35

Following the data analysis, the research team undertook a process of member checking to validate that the emergent codes and categories accurately represented the perspectives of the interviewees. This crucial step ensured the credibility and trustworthiness of the findings by allowing interviewees to confirm or offer refinements to the interpretations derived from their interviews.

### 2.2 Checklist validation

#### 2.2.1 Participants

The sampling frame encompassed one middle school in Fujian Province and two high schools—one located in Fujian Province and the other in Jiangsu Province. These institutions had established collaborative partnerships with the research team. Employing a stratified random sampling approach, a total of 12 classes were selected—two classes from each grade, spanning Grades 7 to 12. Subsequently, participants were recruited through cluster sampling from these preselected classes throughout September 2024. The final sample size amounted to 453 students (with an effective sample rate of 87.6%). Cases were excluded if participants selected identical options across ASAEC or provided incorrect responses to two lie-detection questions, comprising 206 middle school students and 247 high school students. The participants’ mean age was 15.31 years (SD = 2.32), with 212 males and 241 females included in the sample.

Data collection took place during the spring semester of 2025 (from March to May). Participants accessed and completed the questionnaire via the Wenjuanxing online survey platform in their respective school computer labs. To ensure the integrity of the data collection process, trained teachers supervised the survey administration and were readily available to clarify any questions students had. On average, participants spent 1,168 s completing the questionnaire.

#### 2.2.2 Instruments

This study utilized ASAEC, which was constructed based on the qualitative part of the research. The checklist underwent an iterative process of drafting, discussion, and revision by the research team. Subsequently, during the member checking process, respondents were asked to share their perceptions of these items. The checklist comprises 35 items (content available in Appendix 2). The checklist comprises more items than the 32 SAEs identified in the qualitative research. This discrepancy arises because certain SAEs necessitated disaggregation into multiple items to ensure their comprehensive representation. The justification for this disaggregation was subsequently validated through internal discussions and member checking. The checklist has a Cronbach’s alpha reliability of 0.960. Details regarding its reliability and validity will be reported in the Results Chapter.

To provide evidence of criterion-related validity, several other variables are gathered to examine their significant correlations. It is believed that AS should be positively correlated with anxiety, insomnia, gastrointestinal discomfort, and academic disaffection, while negatively correlated with life satisfaction, self-perceived health, and academic engagement.

The General Anxiety Disorder-7 Scale, consisting of seven items, was employed. In the research, the scale demonstrated a Cronbach’s alpha of 0.948. The Satisfaction With Life Scale, a 5-item measure, was also used. In the research, Cronbach’s alpha was 0.878. Its Chinese adaptation was previously validated (Zeng et al., 2013; Cai et al., 2008).

Furthermore, items from the Engagement and Disaffection in the Classroom Scale (Skinner et al., 2008) were utilized, specifically the subscales of academic behavioral engagement and academic behavioral disaffection, each containing five items. In the research, Cronbach’s alphas were 0.901 and 0.907, respectively.

To assess students’ perceived health status, a 5-point Likert item was used, asking, “How would you rate your health compared to your peers?” Two items were used to evaluate insomnia and gastrointestinal discomfort: “Have you had difficulty falling asleep in the past 7 days?” and “Have you experienced stomachaches in the past 7 days?”

The research, considering simplicity and acceptability, used three self-reported survey items to reduce respondent fatigue. The primary purpose of this study was to provide evidence for the criterion-related validity of the ASAEC using these variables, rather than to precisely measure and explore them. Therefore, we believe these items are sufficient to provide preliminary insights. A demographic questionnaire was also administered to collect data on age, gender, and grade level.

#### 2.2.3 Data analysis

In the research, IBM SPSS Statistics 25.0 was used to calculate Cronbach’s alpha coefficients, means, and standard deviations for each variable. A Cronbach’s alpha value exceeding 0.8 was considered indicative of reliable measurement, ensuring that the checklist consistently captured the intended constructs. Data on the means and standard deviations of variables serve as essential evidence for future replication and application of this research.

Previous empirical studies have established the commonsense that academic stress is positively correlated with anxiety, insomnia, gastrointestinal discomfort, and academic disaffection, while negatively correlated with life satisfaction, self-perceived health, and academic engagement. To assess the criterion-related validity of ASAEC, the researchers computed the correlation coefficients among variables using SPSS 25.0. Criterion-related validity would be supported when correlations aligned with theoretical expectations and achieved statistical significance (*p* < 0.05).

Exploratory factor analysis was conducted using SPSS 25.0 to assess the construct validity of the scale. The Kaiser–Meyer–Olkin (KMO) measure of sampling adequacy (≥0.7) and a significant Bartlett’s test of sphericity (*p* < 0.05) confirmed the appropriateness of factor analysis. The number of factors was determined via their eigenvalues, a robust data-driven method. When the ratio between the first eigenvalue and the second eigenvalue exceeds 3, it indicates the retention of a single factor, supporting the single-factor structure of the checklist to measure academic stress. This finding aligns with theoretical expectations and provides evidence of construct validity.

To examine the single-factor structure of the items, a confirmatory factor analysis was conducted using Mplus 8.3. If all the path coefficients are significant and greater than 0.4, then the items have a good fit to the model. Model fit was evaluated through multiple indices: a root mean square error of approximation (RMSEA) < 0.08 and a standardized root mean-square residual (SRMR) < 0.08 were considered indicative of acceptable fit, since the two indices reflect the absolute fit. A comparative fit index (CFI) of greater than 0.9 and a Tucker-Lewis index (TLI) of greater than 0.9 indicate an excellent fit, as they are relative fit indices that reflect the model’s significant improvements compared to the baseline model.

A single-factor, two-parameter item response theory model for ASAEC was constructed using the mirt 1.44.0 package in R 4.4.1. Model fit was evaluated through multiple indices: RMSEA < 0.08, SRMR < 0.08, CFI > 0.9, and TLI > 0.9, which were considered indicators of model fit, further validating the scale’s construct validity. Item quality was assessed based on discrimination parameters (greater than 0.4), average information values (greater than 0.5), and standardized mean square (MNSQ) fit statistics for outfit and infit (ranging from 0.8 to 1.2). These criteria confirmed that items effectively differentiated participants across trait levels, provided substantial measurement information, and aligned well with the sample, indicating high-quality items.

## 3 Results

### 3.1 CIT

Figure 3 illustrates the main findings, where the abstract categories are represented in the pie chart, and the corresponding SAEs are displayed on the corresponding arcs. The color of the SAEs indicates the number of times they are mentioned in 540 critical incidents, with red denoting>40, orange denoting 20–39, yellow denoting 10–19, and green denoting <10. The social actors and interactions corresponding to each SAE are presented at the left and right sides of the colorful words, which are in black signals and initials, with arrows for direct interactions, tabs for mediated interactions, circles for observing interactions, stars for distal interactions, P for parents, C for classmates, F for friends and other non-classmate peers, T for teachers, M for media, K for kinsmen, Ng for neighbors or those who live around, Nt for netizens, and S for self.

#### 3.1.1 Enrollment

**Being arranged for an academic goal** primarily stems from direct interactions with parents, followed by direct interactions with teachers, kinsmen, and neighbors. For example, A9 stated, “*My parents repeatedly reminded me thousands of times, saying, ‘You must get into a good school.’ The word is “MUST”; it made me feel that getting admitted became my mission, something I had to achieve. If I failed to do so, it felt like I could not meet their expectations, and this gave me a heavy burden.*” Similarly, B14 shared, “*Kinsmen would often ask about my academic progress and suggest that I get into this or that kind of university.*” Such expectations from others regarding students’ academic performance can lead to AS.

**Difficult to be admitted** mainly arise from direct interactions with news media, followed by direct interactions with teachers. For instance, A9 explained, “*Online media often publish data. When I see these statistics, I compare myself to them and figure out where I stand. If I find myself in an unfavorable position—like, if there are 490,000 test-takers this year and the admission rate is just over 30%—this comparison gives me immense pressure.*” Media reports on reference groups make A9 feel that the information directly applies to him, creating AS through direct interaction. Similarly, A20 noted, “*Teachers often introduce to students how competitive the society is and how low the admission rates are, which increases the pressure on children.*” The essence of this SAE is the information about the difficulty of gaining admission.

**Being criticized for low scores** comes from direct interactions with parents and teachers. For example, B16 shared what a teacher once said to him, “*Everyone else is making progress; only you are just standing still—or even going backward,*” which caused him to feel stressed. Similarly, C11 mentioned that when his parents said, “*Why are your grades so bad?*” it also contributed to his AS.

**Being worried for academic achievements** mainly originates from direct interactions with parents. For instance, B10 quoted his parents saying, “*What can you do after you cannot get enrolled?*” He believed such comments triggered AS. Worry can be seen as a softened form of criticism.

**Academic achievements as conversation piece**, arising from direct interactions with kinsmen, neighbors, or people living nearby. For instance, B20 mentioned, “*Neighbors would greet me by starting with questions about my academic achievements,*” which created a sense of AS. Similarly, A14 noted, “*At family gatherings, kinsmen and friends of my parents would ask about my final exams or monthly test scores. They’d also ask if I ranked high in my class and grade.*” These conversational practices, often framed as casual inquiries, can induce AS among middle and high school students.

**Discussing or flaunting the score** stems from direct or observing interactions with classmates or friends. For example, B16 recounted how a classmate once remarked, *“I did not even study much, but I still got this score,*” which caused him to be stressed. C17 observed that “*Friends often talk about how far ahead they have studied and compare their grades,*” and he felt stressed when he observed such conversations.

**Having academic goals** is a form of distal interaction. A7 stated, “*Children’s pressure is closely tied to their living environment. Their self-perception and value system shape their expectations of themselves, which, in turn, increases their stress.*” Similarly, A10 shared, “*I’m still some distance away from achieving my expectations for myself, and that creates pressure.*” These academic expectations are the internalization of long-term external discipline rooted in academic goals.

**Unbalance of effort and gain** is another form of distal interaction. A11 noted, “Some students want to change their destiny and strive for a better future, so they aim for breakthroughs in their studies. However, the learning strategies may not be effective, which results in big psychological pressure.” A9 added, “I’ve put in so much effort, but in the end, it feels like it’s twice the effort for half the result.” This SAE reflects “having an academic goal” in terms of outcomes.

Academic achievements determine whether a student can be enrolled in high school or university. External factors significantly influence whether students can advance to the next level of education, prompting them to set academic achievement goals for themselves. When these goals are not met, students face criticism and worry about their grades, compounded by constant messages about the difficulty of exams and low admission rates. These circumstances collectively create an AS for students centered around the theme of “enrollment.”

#### 3.1.2 Rivalry

**Being compared to peers** arises from mediated interactions with parents, kinsmen, family friends, neighbors, or others living nearby. For example, A11 shared, “*At family gatherings, kinsmen would ask about my grades. They’d say, ‘How did you do compared to [Another kid]? Which one of you did better?*” Similarly, A15 mentioned, “*During the Chinese New Year, when we visit kinsmen and friends, they’ll definitely ask how the final exams went. If they also have kids at a similar stage, the comparison is inevitable. Parents care a lot about saving face, so the pressure they feel gets passed on to us.*” This SAE stems from being compared to peers by parents, kinsmen, family friends, and neighbors, connecting competitive ones. Such AS reflects the academic rivalry under the current education system.

**Being set an academic example modeling** comes from observing interactions with media or mediated interactions with teachers. For instance, A16 explained, “*News and online media often report on particularly outstanding students, and it makes me feel like the gap between me and others of the same age is huge. That puts a bit of pressure on me.*” Similarly, A14 noted, “*After every exam, when the results come out, teachers will praise certain kids who have made progress or have been doing really well lately.*” Whether through media glorifying exceptional students or teachers praising high-achieving or improving students in front of others, these interactions set academic example models for students, thereby creating AS.

**Public ranking**: This is a form of bridging interaction from teachers. For example, A6 shared, “After every exam, the teacher announces everyone’s grades and ranks us in the class.” The essence of this practice lies in teachers providing students with information that encourages them to compare their grades with those of their peers.

**Coming to compare with me** comes from direct or observing interactions with classmates or friends. For instance, C15 recounted a classmate saying to him, “*I managed to get into the top 10 in the grade—what about you?*” Similarly, A6 noted, “*Friends and classmates often ask each other how they did on this exam or the previous one. They’ll also talk about how other classmates scored, especially if someone scored higher than me.*”

**Others learn but not tell me** stems from observing interactions with classmates. For example, A8 commented, “*The pressure from classmates comes mainly from involution. Students often feel like everyone is secretly studying, so they need to work harder as well. Or they might even feel jealous of those who are secretly putting in extra effort.*” Similarly, B18 said, “*They’re studying behind my back*.” This creates a sense of a toxic competitive environment, which contributes to AS.

**Comparing to others** is a form of distal interaction. For instance, A2 shared, “*When I’m solving problems, I sometimes realize I’m not as good as my classmates, which makes me feel a bit anxious.*” A9 added, “*When I see that a neighbor’s child has successfully been admitted to their dream school, and I know we started at a similar level, I cannot help but think, ‘What if I do not get admitted?’ Or, if a classmate who used to be worse than me now achieves better scores, I feel like I’m falling behind.*”

External forces, competitive benchmarks, and reference groups often compare middle and high school students to their peers. This fosters an atmosphere of rivalry among students. In such an environment, students internalize these comparisons, leading them to actively compare themselves with others. These circumstances create AS around the theme of “competition.”

#### 3.1.3 Career

**Life prospects** arise from direct interactions with kinsmen and parents. For example, C14 recalled a kinsman saying to him, “*Work hard—we are counting on you to help us get rich!*” Similarly, A2 shared, “*My parents have high expectations for me. They hope I can have a great future, and that makes me feel quite stressed.*” When others express expectations about students’ future life prospects, it can trigger AS among secondary school students.

**Emphasizing the importance of learning** originates from direct or observing interactions with media, parents, teachers, or kinsmen. For instance, B1 mentioned, “Videos online about people living in poverty because they did not get into college,” which made him feel AS. These media messages, framed negatively, imply that only good grades can lead to a prosperous future, prompting students to imagine themselves in those scenarios and thereby feel stressed. Similarly, C4 observed a kinsman criticizing their cousin, saying, “*With grades like that, he can only go to a vocational school—it’s not even worth attending.*” C4 observed this criticism, recognizing the social stigma associated with poor academic performance, which in turn caused AS.

**Difficult to get a job** often stems from direct interactions with the media or netizens. For example, A7 stated, “*There’s this general fear of the unknown—whether I’ll be able to find a job or not, and the lack of clarity regarding career paths. News and online media often report on college admissions, recruitment, and especially the intense competition for civil service exams, all of which create academic pressure for students.*” Similarly, C13 quoted an Internet user’s warning: “*If you do not work hard now, you will not even have the chance to do manual labor. Wishing alone will not help—you need to take action.*” This warning made C13 feel stressed about his academic performance.

**Setting career example modeling** peer models influence AS in other adolescents through the mediation of parents, as B17 said, “*My parents constantly praise other kids of my age for their extraordinary achievements.*” Peer models are often reported in the media, so they interact with other adolescents through observation to emulate AS, as B4 mentioned, “*News reports about students getting admitted to prestigious universities and contributing to the country.*” External information sets these success stories as benchmarks of life achievement, emphasizing the importance of learning.

Academic achievements are portrayed as playing a vital role in shaping future life prospects. According to societal expectations, students without credentials face difficulties finding jobs, while current economic conditions and intense competition suggest that students with poor academic performance are likely to earn lower incomes. Only those with exceptional academic success are perceived to have bright futures. These narratives, reinforced by societal messages and interactions, form a core logic that places significant emphasis on “career,” making it a key theme through which students experience AS.

#### 3.1.4 Work hard

**Finishing more academic tasks** arises from direct interactions with teachers. For example, B15 stated, “*The teacher gives us way too many outlines, and I cannot get through them all,*” while A20 shared, “*The curriculum in the first year of high school is overloaded. There’s not enough time to allocate every day, so I’m constantly scrambling, and I only manage to learn things halfway.*” Excessive academic tasks make it difficult for students to meet the set goals, contributing to their AS.

**Investing more time learning** comes from direct interactions with parents, netizens, or the media. For instance, A9 said, “*Every time I take a break, my parents keep reminding me over and over to study hard.*” C13 quoted online media saying, “*Studying is tough, but persistence is cool. If you want to achieve your dreams, you must never give up!*” Similarly, C15 quoted netizens commenting, “*Good grades come from hard work.*” These “commands” make students feel guilty about resting or entertaining, leading them to believe that dedicating all their time to studying is the only way to meet external expectations.

**Being criticized for not working hard** stems from direct interactions with parents. For example, C11 quoted their parents saying, “*Why are you always playing and not doing your homework?*” Similarly, B20 shared, “*When I play during the holidays, my parents always think I’m not working hard enough. They keep saying I’m fooling around and not reading my books.*” This reflects a negative expression of the demand to “invest more time learning.”

**Chatter for learning** comes from direct interactions with parents. For instance, C5 said, “*My parents always talk about studying—it’s all they ever say,*” while C12 noted, “*My parents keep repeating things over and over. They nag a lot.*” This constant chatter is a repetitive action of “invest more time learning.”

**Lack of time** arises from direct interactions with teachers and the media. For example, A10 shared, “*Teachers make study plans based on the college entrance exam timeline, and the schedule keeps getting tighter. It makes me feel a sense of urgency.*” A6 added, “*I’ve seen news online about the college entrance exam—like how the test papers are already being transported or the countdown to the exam.*”

Teachers overload students with academic tasks and create an atmosphere of urgency by emphasizing tight schedules. Parents, through constant urging, chattering, and criticizing, demand that their children devote more time to studying, while the media amplifies the AS. This combination of unlimited external demands for students to “work hard” leaves little room for rest and entertainment, creating significant AS for students.

#### 3.1.5 Intelligence

**Talent being devalued** is a direct interactions with parents and teachers. For example, as C1 stated, “*Parents belittle their children, frequently criticizing and undermining their confidence. Regardless of how well the child performs academically, they always convey the attitude that ‘you are not good enough.’* “This type of interaction imposes AS on students. Similarly, C4 quoted a teacher saying, “*Why are you so dumb? You cannot do anything right.*” Such devaluation of intelligence lowers students’ academic self-efficacy, making them believe they are less capable of succeeding in further education or employment compared to others, thereby exacerbating AS.

**Taunts about academic achievements** often originate from direct interactions or observing interactions with classmates, friends, or netizens. For instance, C3 quoted an online comment: “*This is very easy, but you still scored so poorly.*” Similarly, C9 mentioned, “*Often joke because a friend has poor grades.*” While these interactions may not directly target the individual himself or the entire group of middle and high school students, observing such comments and behaviors can lead students to experience AS.

**Being predicted low score** is a direct or observing interaction among classmates. For example, A9 shared, “*After one exam, when my Chinese score was better than my average achievement, I casually asked about the top score in the grade. Then a classmate said, ‘Do you really think you could be the top scorer?’ This kind of remark made me doubt myself.*” Similarly, B14 noted, “*Classmates say things like, ‘I’m afraid I will not pass or will do poorly,’ spreading negativity.*” Classmates serve as an important reference group for middle and high school students. When students predict poor academic performance for themselves or others, it can trigger self-doubt about their academic abilities, leading to AS.

**Working hard but cannot follow others** is an observing interaction with classmates. For instance, B18 said, “*I see my classmates making progress, but I remain stagnant.*” Similarly, A17 noted, “*Some comparatively outstanding students seem to study with ease. Meanwhile, others may put in great effort but are still unable to achieve the same outcomes. I think this can cause pressure for students.*”

**Doubting my own talent** is a form of distal interaction. For example, B9 mentioned that perceiving himself as “*dumb*” can lead to AS. Similarly, B7 pointed out that “*students lacking direction and confidence*” can also result in AS.

Possessing a certain level of intelligence is a prerequisite for academic achievement. When external information causes students to doubt whether their intelligence is sufficient for academic success, it generates AS. All of these interactions revolve around the central theme of “intelligence.”

#### 3.1.6 Social

**Threats** are direct interactions with parents or teachers. For example, C6 quoted a parent saying, “*You’re always on your phone, not studying. Next time, I’ll take it away.*” Similarly, C1 mentioned, “*Teachers physically punishing or scolding children*” can lead to AS. Since parents and teachers have authority over students’ lives, they can threaten to disrupt students’ routines. Such disruptions to students’ cognitive consistency can result in AS.

**Moral blackmail** often stems from direct interactions with parents. For instance, C8 shared that parents emphasize “*the sacrifices they have made and the money they have spent*” for him. C2 quoted his parents saying, “*If you do not study harder, how could you face us?*” Rooted in Confucian values of filial piety, Chinese culture enables parents to exert pressure on their children through moral obligations.

**Social conflict in school** with classmates or friends arises in direct interactions. For example, C1 mentioned, “*Classmates exclude or mock*,” and C2 noted, “*There is not a single classmate in my class who likes me.*” Since middle school students spend the majority of their time in classrooms for academic purposes, social issues that occur in this setting are often tied to academic activities. As a result, some students believe that social conflicts within the school contribute to AS.

**Deciding social attitude based on score** stems from direct interactions with teachers, classmates, or netizens. For instance, B5 stated, “*Teachers decide to pay attention to students or not based on their grades,*” while C5 added, “*Teachers evaluate students’ overall worth based on their academic performance*.” C9 shared that “*classmates secretly mock, isolate, or bully those with poor grades,*” and C3 noted, “*Online users equate poor academic performance with poor character.*” There is a stereotype equating good grades with moral integrity. This perception came from historical connections and persists today, making students with lower grades more likely to encounter social difficulties, which in turn leads to AS.

The attitudes of people around students are often shaped by academic performance. More directly, parents and teachers may use threats or moral blackmail to push students to work harder academically. As a result, students feel compelled to study harder to avoid social conflicts caused by poor performance or insufficient effort. However, students may also face bullying at school, which could discourage them from continuing their studies. These “social” issues, caused by academic activities, are a significant source of AS.

### 3.2 Checklist validation

The internal consistency of ASAEC was excellent, with a Cronbach’s alpha coefficient of 0.960, indicating high reliability. Descriptive statistics and bivariate correlations among study variables are shown in Table 3. All correlations followed the hypothesized directions and were statistically significant (*p* < 0.05), providing initial support for the criterion-related validity of the checklist.

**Table 3:** Mean, SD, and correlation matrix.

Variables	1	2	3	4	5	6	7	8
1	1							
2	0.495*	1						
3	−0.238*	−0.304*	1					
4	−0.229*	−0.299*	0.274*	1				
5	0.355*	0.519*	−0.248*	−0.287*	1			
6	0.283*	0.378*	−0.240*	−0.172*	0.509*	1		
7	−0.097*	−0.152*	0.355*	0.169*	−0.163*	−0.139*	1	
8	0.341*	0.387*	−0.303*	−0.227*	0.297*	0.220*	−0.349*	1
M	98.161	11.793	20.161	3.470	1.550	1.610	18.570	12.678
SD	23.953	4.925	5.787	0.916	0.662	0.598	3.632	4.501
range	35–175	7–28	5–35	1–5	1–3	1–3	5–25	5–25

1. academic stress, 2. anxiety, 3. life satisfaction, 4. perceived health, 5. insomnia, 6. gastrointestinal discomfort, 7. academic engagement, 8. academic disaffection.

*p < 0.05.

EFA was conducted to examine the underlying structure of the checklist. The KMO measure of sampling adequacy was 0.959, and Bartlett’s test of sphericity was significant (*χ*^2^ = 9,881.996, df = 595, *p* < 0.001), indicating the appropriateness of factor analysis for the data. The eigenvalue decomposition revealed a dominant first factor (eigenvalue = 15.129), accounting for 43.226% of the total variance, with the second factor (eigenvalue = 2.270) explaining only 6.486%. The eigenvalue ratio of 6.665 (i.e., 15.129/2.270 > 3) strongly supported a single-factor structure for the checklist. These results suggest that ASAEC effectively captures a single overarching construct of academic stress among adolescents.

**Figure 4:**
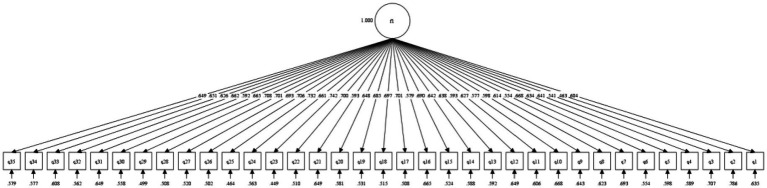
CFA model fit.

The CFA model also indicated that the single-factor structure is acceptable since all paths in the model are significant and their values are greater than 0.4 (see Figure 4). The absolute model fit indices demonstrated acceptable results (RMSEA = 0.071 < 0.08, SRMR = 0.066 < 0.08), indicating that the constructed model fits well. However, the relative fit indices do not yield better results (CFI = 0.836 < 0.9, TLI = 0.824 < 0.9), indicating that the single-factor model does not demonstrate a significant superiority compared to the baseline model.

Several details regarding the EFA method warrant discussion from the perspectives of psychometrics and statistics. However, the research did not intend to explore the controversies surrounding these methods. Therefore, principal component analysis is reported since it is the earliest and most widely recognized approach among researchers. It is worth noting that the results remain similar when alternative extraction and rotation methods are applied; the single-factor structure will still be accepted based on the eigenvalue criterion. The *χ*^2^/df = 16.608 > 3, which may indicate that the model is insignificant and potentially has issues with data fit. However, when the sample size exceeds 200, this statistic can increase the likelihood of Type I errors. Therefore, it is believed that other fit indices should be considered, which will be further elaborated upon in the subsequent IRT model analysis.

In the IRT analysis, the single-factor 2PL model demonstrated an acceptable fit to the data: RMSEA = 0.076, SRMR = 0.076 (both ≤ 0.08, which is the threshold for a reasonable fit), TLI = 0.907, and CFI = 0.905 (both ≥ 0.90, indicating adequate model correspondence). Collectively, these indices support the structural validity of the ASAEC.

Reliability estimates from the IRT model were exceptionally high, with an empirical reliability coefficient of 0.966 (reflecting sample-specific measurement consistency) and marginal reliability of 0.967 (generalizable to the target population), providing robust evidence of internal consistency. Given that over 95% of participants fell within the trait score range of *θ* = −2 to +2 (covering the majority of the adolescent population), the average information statistics were calculated within this practical interval. The total test information function reached 27.197, yielding a very small standard error of measurement (SE = 0.192). This level of precision indicates that the checklist is sufficiently reliable for high-stakes applications such as educational evaluation and clinical diagnostic purposes.

Item-level analyses revealed strong psychometric properties for all 35 items in the IRT model (see Table 4). Discrimination parameters ranged from 1.007 to 2.236, exceeding the minimum threshold of 0.4 for effective trait differentiation. Average item information values remained above 0.3 across the operational θ range, ensuring consistent measurement precision. Fit statistics for individual items—including outfit and infit mean square standardized (MNSQ) values—ranged from 0.927 to 1.144, well within the acceptable interval of 0.8–1.2, confirming no significant misfitting items. These results collectively demonstrate that the checklist items function appropriately across the targeted adolescent population.

**Table 4:** Item quality.

Items	Discrimination	Information	Outfit	Infit
1	1.558	0.641	1.032	1.007
2	1.077	0.331	1.101	1.045
3	1.262	0.451	1.054	1.022
4	1.627	0.702	1.061	0.988
5	1.615	0.694	1.085	0.992
6	1.769	0.803	0.927	0.954
7	1.355	0.523	1.076	1.044
8	1.483	0.619	0.988	1.001
9	1.511	0.622	1.031	1.012
10	1.428	0.563	0.998	1.002
11	1.541	0.663	0.984	0.993
12	1.461	0.577	0.991	0.987
13	1.657	0.690	0.970	0.987
14	1.713	0.752	0.988	0.982
15	1.923	0.998	1.026	1.021
16	1.416	0.571	1.012	1.002
17	2.050	1.112	0.985	1.011
18	1.884	0.962	0.993	1.000
19	1.840	0.937	1.005	1.012
20	1.736	0.808	1.021	1.025
21	1.446	0.580	1.036	1.011
22	1.934	0.974	1.020	0.989
23	2.236	1.245	0.965	0.957
24	1.702	0.712	0.933	0.948
25	2.214	1.212	0.974	0.980
26	1.991	0.997	0.959	0.996
27	1.951	0.974	0.985	1.015
28	2.010	1.040	0.997	1.006
29	2.096	1.139	1.144	0.992
30	1.825	0.878	1.089	1.024
31	1.376	0.531	1.043	1.022
32	1.709	0.805	0.984	1.004
33	1.548	0.631	1.013	1.019
34	1.646	0.700	0.962	0.984
35	1.682	0.757	1.032	1.028

## 4 Conclusion

### 4.1 Q1: common SAEs in the digital and urban context

The common SAEs experienced by adolescents are shown in Figure 3. Most SAEs recognized in the research have been mentioned in prior studies. However, the research yields some new findings, particularly regarding SAEs in digital and urban contexts.

There are some new SAEs related to mobile Internet. The information “emphasizing the importance of learning”, “difficult to get a job”, and “difficult to be admitted” comes from media and netizens. Netizens and media would suggest adolescents about “investing more time learning”. Netizens would also “taunt on academic achievements” and “decide social attitude based on score”. And the information “being set academic example modeling”, “lack of time” come from media. The condition differed from early findings that the Internet merely exacerbates the social conflicts between adolescents and their families and friends (Boyle, 2020; Cusato, 2022). In research, social media SAEs are often from strangers, and they tend to focus more on news and comments related to learning.

Some new SAEs are associated with the urban community. Several statements suggest that kinsmen and neighbors “arise being arranged for academic goal”, kinsmen, neighbors, or people living nearby arise “academic achievements as conversation piece”, kinsmen, family friends, neighbors, or others living nearby arise “being compared to peers”, kinsmen arise “life prospects”, the information “emphasizing the importance of learning” can also come from kinsmen. These findings provide further evidence that the urban community plays a significant role in shaping adolescents’ AS.

### 4.2 Q2: quantitative examination of the common SAEs

All SAEs related to the urban community and online interactions are significant in the ASAEC developed according to the qualitative research phase. The quantitative phase provides strong evidence for this: in the CFA model, all items have significant path coefficients greater than 0.4, indicating that, for a typical cluster of adolescents, all items are strongly connected to the academic stress indicator. In the IRT 2PL model, all items exhibit good fit indices from multiple perspectives, indicating that they can effectively discriminate between low- and high-stressed adolescents. All items can provide useful information rather than useless noise, and all items have good item-person fit. The quantitative evidence from item fit indices in CFA and IRT examines the common SAEs applicable to a broad population, supplementing the detailed qualitative data.

### 4.3 Q3: weak ties are also attributed to SAEs

The neighborhood and online society contribute substantially to AS. In the research, neighbor interactions were mentioned 206 times (including neighbors, those who live around, kinsmen, friends, and non-classmate peers), and online society was mentioned 82 times (referring to media and netizens), indicating that weak ties participated in over half of the critical incidents (53.3%). As anticipated in the research, students’ AS should take weak ties into account; urbanization and the rise of mobile Internet provide opportunities for weak ties to influence AS. According to Granovetter (1973), although weak ties are more dispersed and less easily detectable, interactions associated with weak ties occur more frequently than those associated with strong ties. The higher proportion of weak ties in the research (53.3% > 49.8%) aligns with the theory.

### 4.4 Q4: mediated interaction and observing interaction can bring AS

In the interaction way dimension of the qualitative research framework, mediated interaction and observing interaction are important in understanding AS. Prior studies have primarily distinguished AS from direct interaction and self-stress. However, in the research, mediated and observing interactions accounted for 34% of critical incidents. Compared to only 6% for distal interactions, the two should be considered as new categories since they have a far greater proportion.

In research, the frequency of interaction across the various layers of the system decreases progressively from proximal to distal levels, aligning with ecological systems theory, which posits that interactions occur less frequently as the distance increases. This finding partially supports the rationale for interpreting self-stress as a distal interaction in this research.

### 4.5 Q5: single-factor structure of the SAEs

There is no dominant dimension when explaining the factor structure of the SAEs. The three dimensions are interconnected in the qualitative phase, where SAE topics involve more than one social actor and interaction way, and vice versa. Similar evidence also appears in the quantitative data—the EFA results showed a single-factor structure and indicated that there is no dominant dimension that can serve as the basis for separating the multiple factors. The conclusion aligns well with the results of CFA and IRT. Since the fit indices of single-factor models are not perfect, it suggests that AS might require a more complicated construct.

### 4.6 Q6: examinations of the checklist

The research examined the ASAEC from four perspectives. The information gathered during the qualitative phase provided content validity for the checklist. The qualitative phase followed the CIT process to gather interview data, which was then analyzed using the CGT method, resulting in 35 SAEs, the sources of the items on the checklist. In the quantitative phase, criterion-related validity was examined, as hypothesized, since AS is positively correlated with anxiety, insomnia, gastrointestinal discomfort, and academic disaffection, while being negatively correlated with life satisfaction, self-perceived health, and academic engagement. Meanwhile, the fit indices in EFA, CFA, and IRT supported the conclusion that the checklist has acceptable structural validity. Finally, the Cronbach coefficient indicated that the checklist has high internal consistency reliability.

## 5 Discussion

### 5.1 Implications

#### 5.1.1 Highlights on new trends of AS

As urbanization increases, more students live in acquaintance neighborhoods, rendering weak ties increasingly important in their lives (Marquez et al., 2024; Song Y. K. et al., 2023). Similarly, the mobile Internet and social media have been popular for a decade; cyber connections should not be ignored when considering students’ academic lives (Moreno et al., 2021; Cusato, 2022). The research filled a gap in exploring their influence, finding that weak ties have an influence on middle and high school students in a manner different from strong ties. The research, in particular, emphasized the influence of weak ties, as well as mediated and observing interactions. These SAEs should also be taken into consideration in academic research, psychometrics, counseling, educational policies, and recommendations to adolescents.

#### 5.1.2 Intertwined dimensions and “self” stress

AS scales lack a clear definition and discussion of their construct taxonomy, instead relying on the EFA method to separate items and assign factor names. This unconscious taxonomy caused chaos in the field. Some scales tended to use the SAE topics (Ang and Huan, 2006; Kaynak et al., 2023) or the social actors (Xu et al., 2010) as their potential criteria for naming the factors, while some even mixed the two (Garcia-Ros et al., 2018; Hosseinkhani et al., 2020). For example, Garcia-Ros et al. (2018) named two factors, “family pressure” and “interaction with classmates,” from the dimension of social actors, while naming two others, “academic overload” and “future-oriented perspective,” from the dimension of SAE topics. Similarly, the category of “self” stress was underdefined, as it can be used as an SAE topic (Sun et al., 2011) or as a social actor (Ang and Huan, 2006; Xu et al., 2010).

The research discussed the issue by summarizing existing literature and introducing a third dimension to explain the dual role of “self” stress from the perspective of ecological systems theory. In the qualitative phase, the research revealed that the three dimensions are intertwined. Such a phenomenon explains why diverse taxonomies emerged in previous literature: starting from different data, various studies attempted to verify that a certain facet is the dominant dimension or to reconcile multiple dimensions to construct models. They selected evidence that supports their arguments to derive seemingly reliable conclusions, but each significant finding revealed only one aspect of the overall picture.

#### 5.1.3 An induction-based scale

Previous studies exploring Chinese adolescents’ SAEs often claim to use inductive methods for item development. However, these studies typically lack rigorous argumentation regarding their empirical inductive processes. They neither present detailed survey data nor provide a transparent narrative of how evidence was used to generate a checklist. Instead, their focus tends to shift prematurely to the subsequent step of examining the psychometric quality of the scale (Ang and Huan, 2006; Sun et al., 2011). As a result, the theoretical foundations underlying these scales remain unverified, leading to a lack of robust evidence for content validity.

Other checklists for Chinese adolescents have relied on psychological theories, expert proposals, research team insights, or so-called literature reviews to generate items (Wang, 2008; Xu et al., 2010; Lin, 2014). However, these deductive approaches lack support from examined inductive evidence. They were based on vague or insufficient inductive evidence, thus failing to produce reliable scales. Moreover, the deductive process itself can introduce issues such as defective items or indicator pollution (Morgado et al., 2017).

This study addresses these shortcomings by providing transparent, evidence-based, inductive procedures for developing ASAEC. It offers stronger empirical support for internal consistency, content validity, criterion-related validity, and construct validity. Additionally, this checklist is more contemporary, encompassing SAEs that arise within the urban and digital contexts shaping adolescents’ lives today. By filling the gap in methodological rigor and ensuring the inclusion of evolving stressors, the ASAEC represents a significant advancement in the field of academic stress measurement.

### 5.2 Limitations

Due to resource constraints, this study did not adopt stratified and phased sampling. All samples were recruited from two towns in China, which means the life experiences reported by participants during the qualitative phase may not be transferable to rural areas and other cultures, and the statistical results derived from the quantitative research phase may lack representativeness for broader populations. Additionally, the interview outline employed in this study could be further elaborated with clarification prompts for participants’ statements—specifically, participants should have been guided to explicitly clarify which social actors and interaction ways were involved in the SAEs they mentioned. Such prompts would have minimized subjective interpretation during the coding process. Finally, to shorten participants’ response time and ensure response efficiency, simplified measurement tools were used to assess some variables, which may have introduced measurement imprecision.

This study failed to adequately address the issues of intertwined dimensions and “self” stress. The evidence from the qualitative phase lacked sufficient depth, as it failed to fully capture participants’ attitudes toward the theory in question. Had the study asked participants whether they believed an SAE might stem from multiple social actors and interaction ways, the qualitative evidence would have been able to address this question more robustly. Furthermore, the proposed three-dimensional structure of this study requires verification using Cognitive Diagnostic Theory (CDT). However, the current number of items was insufficient to accurately fit a model of such high complexity. This limitation limited the ability of the quantitative findings to substantiate the generalizability of the three-dimensional structure.

### 5.3 Future directions

To prevent adolescents from experiencing increasing AS in the era of urbanization and mobile Internet, we should advise them to consciously manage their social relationships and mobile device use, keeping the frequency and intensity of exposure to SAEs within acceptable ranges. Additionally, adolescents need to establish the belief that the purpose of learning is to enhance their lives and work. They should critically assess the increased information in competition and comparison in the new era and use cognitive reappraisal to mitigate the impact of new stress and anxiety events (SAEs). Beyond these recommendations for adolescents, we should also update the psychological measurement tools for AS, standardize processes for academic-related counseling, and refine educational policies for managing student AS. The effectiveness of these measures requires further research.

One interesting finding is the moral blackmail SAE. This is a direct interaction with parents, making it easy to be recognized. However, it has never been mentioned in prior research. Thus, it should be generalized with caution. And this SAE should be further examined and discussed in future research.

The three-dimensional framework may enhance individuals’ perceptions of middle and high school students. It has the potential to improve the AS scale, help counselors better understand the AS of students, and provide policymakers with insights into measures to manage AS. However, this study, unfortunately, did not provide sufficient evidence to support the theory. Relevant interview evidence can be collected to robustly support the theory. If the number of items in the checklist is increased, a CDT model could be established to more accurately validate this hypothesis. This is a direction for improvement considered in this study.

## Data Availability

The raw data supporting the conclusions of this article will be made available by the authors, without undue reservation.
