# Supplementary material for: Exploring adolescent academic stress in the digital and urban age: a mixed-methods study from CIT to checklist validation
Source: Front Psychol. 2025 Nov 12;16:1692113. doi: 10.3389/fpsyg.2025.1692113 (PMC12646907; doi:10.3389/fpsyg.2025.1692113)
Supplement: Supplementary file 1 [file Data_Sheet_1.docx]

**Appendix 1 interview outlines**

The purpose of Q1-Q3 is to arise the participants' memories about students' academic life, prepare well for the important Q4. Then Q5-Q7 is to arise the participants' memories about students' social interaction, prepare well for the important Q8. Participants' answers to Q4 and Q8 (as well as their following probes) will be coded for further analysis.

1. What everyday activities do you (or your students/ your child) do from waking up to going to bed?

2. What activities do you (or your students/ your child) do from waking up to going to bed in weekends and holidays?

3. Do you think you (or your students/ your child) are under a high level of AS? (probe if the answer is too simple: Why do you think so?)

4. Who give you (or your students / your child) AS? (Whoever the participant answer, probe how? What do he/she do to generate AS? Then probe are there any others? If participant answer another people, keep probe the same. When the participant said no other stressors, probe if the participant did not mention anyone of the following, probe whether do you think [role] can give you AS, why?[roles: parents, teachers, classmates, students themselves])

5. [This question is only for parent and student participants] Which kinsman and friends are familiar with your whole family

6. How is the frequency for you (or your students / your child) to use e-devices? What do you (they) do with e-devices?

7. Do you (they) see videos, news or social hotspots on the Internet? Do you (they) browse information about learning on the Internet?

8. Who else give you (or your students / your child) AS? (Whoever the participant answer, probe how? What do he/she do to generate AS? Then probe are there any others? If participant answer another people, keep probe the same. When the participant said no other stressors, probe if the participant did not mention anyone of the following, probe whether do you think [role] can give you AS, why?[roles: students' friends outside the class, neighbors, kinsman of parents, strangers in the Internet, news media, Internet self-media])
