# Supplementary material for: Exploring adolescent academic stress in the digital and urban age: a mixed-methods study from CIT to checklist validation
Source: Front Psychol. 2025 Nov 12;16:1692113. doi: 10.3389/fpsyg.2025.1692113 (PMC12646907; doi:10.3389/fpsyg.2025.1692113)
Supplement: Supplementary file 2 [file Data_Sheet_2.docx]

**Appendix 2 academic stress checklist**

Options: 1. No such experience; 2. The experience does not make me feel stressful; 3. The experience makes me feel a little stress; 4. The experience makes me feel a lot of stress; 5. The experience make me feel extremely stressful and I cannot stand it.

Each item scores from 1 to 5, and the total score range from 35 to 175.

1. My parents expect me to take exams and be enrolled.
2. Kinsmen or friends of parents expect me to get into an ideal school.
3. News about fierce competition, low admission rates, and difficulty finding jobs.
4. My parents criticize me for poor grades.
5. My teachers are dissatisfied with my grades.
6. My parents are worried about my academic future.
7. Kinsmen or friends of parents discuss my grades.
8. Classmates or friends talk about getting good grades effortlessly.
9. I'm dissatisfied with my academic performance.
10. I can't achieve the goals I set for myself.
11. My parents compare me to children in other families.
12. Learning about excellent role models online and in real life.
13. Rankings are announced after exams.
14. Friends ask about my grades.
15. Classmates compare their grades with mine.
16. Peers around me are secretly learning.
17. I feel like I'm not learning as well as those around me.
18. My parents often nag me, hoping I'll become an outstanding person.
19. The saying "grades determine everything, only learning leads to a good future."
20. Media and parents set examples of those with promising futures.
21. Teachers assign too many or too difficult study tasks.
22. My parents say I don't study hard enough.
23. My parents always nag me to study.
24. Feeling that exams are getting closer and closer.
25. My parents or teachers think I can never learn.
26. Classmates or friends believe that students with poor grades aren't smart.
27. Seeing other students being ridiculed for poor grades.
28. Classmates' discussions affect my confidence in learning.
29. I see classmates achieve good grades, while I remain unchanged.
30. I feel I don't have a talent for learning.
31. Parents or teachers threaten to punish me (e.g., confiscate my phone, beat me).
32. My parents say they've sacrificed a lot for me, and I'd let them down if I don't study hard.
33. Being excluded or ridiculed by other classmates.
34. Evaluating a person's character based on their grades.
35. Some people decide how to treat others based on their grades.
